# Immigration status as a determinant of health information-seeking behavior among undergraduates of color at an urban commuter college

**DOI:** 10.34172/hpp.2022.38

**Published:** 2022-12-10

**Authors:** Vincent Jones II, Sungwoo Kim, Apeksha H. Mewani, Erin T. Jacques, Mary-Andrée Ardouin-Guerrier, Shyanne Huggins, Corey H. Basch

**Affiliations:** ^1^Department of Health and Human Performance, York College, The City University of New York, Jamaica, NY 11451, USA; ^2^Department of Human Development, Teachers College, Columbia University, New York, NY 10027, USA; ^3^Department of Health and Behavior Studies, Teachers College, Columbia University, New York, NY 10027, USA; ^4^Health Promotion Center, York College, The City University of New York, Jamaica, NY 11451, USA; ^5^Department of Public Health, William Paterson University, Wayne, NJ 07470, USA

**Keywords:** Health disparity, Minority and vulnerable populations, Information seeking behavior, Immigration, Social media, Health promotion, Universities, Students, Social determinants of health, Urban health

## Abstract

**Background**: Health information-seeking behavior (HISB) of college students is of importance due to health information inconsistencies at a time when personal independence and concurrent personal health decision making may be increased. Currently, there is a dearth of research about the HISB of college students, especially from diverse backgrounds. Therefore, the purpose of this study was to identify and describe the sources college students use when engaging in HISB; and to explore associations between HISB and demographic factors.

**Methods:** This cross-sectional study was conducted with undergraduate students at a diverse, public institution in New York City. The sample was drawn from an institutional social science research pool and asked to complete a survey on HISB. A total of 226 respondents completed the survey.

**Results:** The believed accuracy of the information found online was positively correlated with related behaviors and beliefs. The number of followers on Instagram proved to be an important mediator of HISB of college students. The number of followers a health information provider has was fittingly positively correlated with the belief that social media is a helpful resource for health information r(233)=0.18, *P*=0.01. Students from families with two or more generations living in the United States accessed health professionals more frequently than students from one or less generation (χ^2^=8.107(2), *P*=0.017).

**Conclusion:** Targeted educational programs designed to increase health information seeking skills, including discernment of information quality should be a priority for college students.

## Introduction

 Studies have indicated that the college years are a vulnerable health period that presents both an opportunity to deliver critical health information to college students,^1-6^ and to better understand their process of gathering information. Health information-seeking behavior (HISB) is the process by which people collect information about health.^7^ HISB is distinguished from health information scanning by being an active, rather than passive process, whereby individuals search for specific information beyond what they are normally exposed.^8^ During HISB, people may not only look for information about their general health, but also health promotion activities, health risks, and illness.^9^

 The literature suggests that college students have an interest in health information, and health information seeking behavior (HISB).^10,11^ Frequently searched health topics among college students included minor health concerns, nutrition and diet, body fitness, and risky health practices.^6^ Statistics from the Pew Research Center show that 89% of college graduates and 70% of high school graduates with access to the internet go online to gather health information.^12^ Social media is an extension of the internet, a channel for seeking health information, and has enabled more possibilities such as connecting people with similar health concerns.^13^ In fact, peer-to-peer interactions and social and emotional support leads consumers to consult social media. In one study, 23% of users on a social media platform reported subscribing to their contact’s personal health narratives.^14^ In another study, college students resorted to social media for health information 87% of the time.^15^ While the Internet is a source of free information, it can also be a major source of misinformationthat has potential to compromise health outcomes.^16^ The ‘information economy’ can contribute to confusion due to “instances of unreliable news reports, glitches in filtering and flagging of information that is valid”.^17^According to the health belief model, adults’ belief in their vulnerability and susceptibility to risks could moderate their intentions to share health information on social media.^18^This could lead to the sharing of poor-quality information about all information, but particularly in relation to pressing issues (e.g. emerging infectious diseases such as COVID-19).

 College students seek health information from multiple sources that may be determined by contextual and situational factors.^5,6,19^ Despite the ubiquity of the Internet and social media, there remains a dearth of research about the HISB of college students, especially from diverse backgrounds. This is an important population to focus on due to the inconsistencies in health education in the US combined with their newfound period of independence, where they are called to discern and apply health information on their own.

## Objectives

 There are two aims of this research: (1) to identify and describe the sources college students use when engaging in HISB; and (2) to explore associations between HISB and demographic factors.

## Materials and Methods

###  Study design

 An unvalidated questionnaire developed by Basch et al^7^ was adapted to assess HISB in a sample of college students. The first section collected demographic information, inclusive of race/ethnicity, gender, age, college major, and income. Categories not represented in the original survey include immigration status, measured by asking the number of generations their families had been in the US, and campus affiliations (i.e. ROTC), commuter status, and number of visits to campus per week. The second section assessed the frequency of seeking information from particular sources. The third section focused on the Internet and social media, in which respondents were asked to recall if they used said resources as sources of health information, and how much time they spent browsing. Then, in another question, they were presented with a menu of popular health sites and asked where they would go first for health information. Next, students were asked if they had self-diagnosed a medical condition and then if they sought confirmation from a medical professional. Finally, the fourth section assessed the extent to which college students assessed the accuracy of information from online sources and shared information.

###  Participants

 The sample was drawn from the institutional social science research pool between fall 2019 and spring 2020 at a public university in New York City. The research pool provides students in certain courses in psychology, social work, sociology and anthropology to participate in research studies on campus. Students in specified courses had the option of receiving course credit for completing a certain number of points if surveys in the pool or could complete an alternative assignment. To take the survey, the participants had to be a student at the College, not have graduated before fall 2019, and able to respond to a survey in English. A priori sample size estimation with maximum degrees of freedom used in the study (df = 4), 95% power, and medium (w = 0.03) effect size indicated that the study would require 207 participants in the sample to reach the target power and effect size. Accordingly, the study’s sample size (n = 226) meets the criteria to generate results with sufficient power. G*Power software was used to calculate this estimate. In total, 253 students attempted the survey, 226 of whom met the inclusion criteria and completed the questionnaire. The questionnaire consisted of four prescreening questions, 18 demographic questions, four binary questions, two fill-ins, and 16 Likert questions that used five- and seven-point scales.

###  Statistical methods

 IBM SPSS Statistics for Windows (Version 27, Armonk, NY) was used to calculate descriptive statistics and perform analyses. A series of correlation analyses were performed among variables to assess the relationship between HISBs and social media usage (refer to Supplementary File 1). Then, chi-square tests of association were performed on HISBs among different demographic traits. An alpha level of α = 0.05 was used to determine significance.

## Results

 The sample represented the broad diversity of the greater Queens, NY area. These demographics are included in Table 1. A total of 3% identified as American Indian or Alaska Native, 25% as Asian, 36% as Black or African American, 1% as Native Hawaiian or Other Pacific Islander, 3% White, 30% Hispanic or Latino, and 9% as multiracial/other. Half (50%) of the participants were freshmen, 18% were sophomores, 18% were juniors, and 14% were seniors. While all academic departments were represented, the plurality of respondents came from the nursing and social work programs, 26% and 24% respectively. Given the availability of online learning modalities, 53% of respondents never commuted to campus. 40% of respondents were the first in their families to attend college in the United States. Further, 48% of participants had been in the United States for one generation or less.

**Table 1:** Demographics

**Demographics**	**N**	**%**
Number of family generations in the United States		
0	35	14.2
1	86	35.0
2	51	20.7
> 2	73	29.8
Total	245	100
Income below / at / higher than median		
< 49 999	174	70.73
50 000~74 999	30	12.19
> 75 000	26	10.56
Total	230	100
Income at low 30%, mid 30%, high 30%		
< 34 999	121	49.2
35 000~99 999	97	39.4
> 100 000	12	4.9
Total	230	100
Race (multiple selection possible)		
Native American	8	3.3
Asian	61	24.8
Black	89	36.2
Hispanic	73	29.7
White	7	21
Other	2.8	8.6
Gender		
Male	40	196
Female	16.3	79.7

###  Temporality

 Hours of day spent browsing the Internet was positively correlated with hours per day on social media (*r* = 0.662, *P* = 0.01). Time spent on the Internet was also weakly correlated with sharing information with family or friends without checking the accuracy (*r* = -0.14, *P* < 0.05), where those who spent longer time browsing were more likely to share the information.

###  Accuracy 

 The believed accuracy of the information found online was positively correlated with related behaviors and beliefs, including gathering online information from multiple sources (*r* = 0.343, *P* < 0.01), sharing with family and friends (0.216, *P* < 0.01), confirming the information with medical professionals (*r* = 0.168, *P* < 0.05), believing the Internet (*r* = 0.320, *P* < 0.01) is a helpful health information resource, and social media as a helpful (*r* = 0.269, *P* < 0.01) and accurate (*r* = 0.275, *P* < 0.01) source of health information.

###  Social media followers and health information seeking behavior

 The number of followers on Instagram proved to be an important mediator of HISB of college students. The number of followers a health information provider has was fittingly positively correlated with the belief that social media is a helpful resource for health information r(233) = 0.18, *P* = 0.01. The number of followers of a health information provider on social media was positively correlated with the belief that information is accurate r(233) = 0.19, *P* = 0.01, in addition to sharing information without checking its accuracy r(235) = 0.32, *P* = 0.01.

###  Gathering information from multiple sources

 Gathering information from multiple sources to ascertain its accuracy was frequently correlated with other variables, namely the belief that the internet is a useful source for health information r(235) = 0.42, *P* = 0.01,belief that the information is accurate r(233) = 0.34, *P* = 0.01 the number of followers of an Instagram information provider r(234) = 0.16, *P* = 0.05., the likelihood of confirming information with a health professional r(235) = 0.34, *P* = 0.01, and sharing information with family and friends without checking its accuracy r(235) = 0.18, *P* = 0.01.

###  Self diagnoses

 Having diagnosed oneself with a medical issue using the Internet was positively correlated with confirming the information with a health or medical professional r(235) = 0.32, *P* = 0.01. The likelihood to confirm information found on the Internet with a medical professional was correlated with actually having seen one r(87) = 0.32, *P* = 0.01.

###  Accessing health and medical professionals

 Chi-square tests indicated the frequency of accessing health and medical professionals across various demographic factors (Table 2). Students from families with two or more generations living in the United States accessed health professionals more frequently than students from one or less generation (χ^2^ = 8.107(2), *P* = 0.017). In terms of ethnicity, Asian students less frequently accessed health and medical professionals, (χ^2^ = 10.541(2), *P* = 0.005). In contrast, black students were more likely to access health and medical professionals (χ^2^ = 6.436(2), *P* = 0.04). They were also more likely to access community centers for health information (χ^2^ = 6.970(2), *P* = 0.031).

**Table 2:** Chi-square tests

**Generation of Famil**y					
		0 or 1*	More than 2*	Total	
Health and Medical Professionals	Always/Often	63	86	149	
	Sometimes	27	15	42	
	Rarely/Never	24	17	41	
Total		114	118	232	
Chi-Square (df), p	8.107(2),.017*				
**Income below/at/higher than median**					
		0~49 999	50 000~74 999	75 000 and higher	Total
Health and Medical Professional	Always/Often	112	15	18	145
	Sometimes	31	4	6	41
	Rarely/Never	27	11	2	40
Total		170	30	26	226
Chi-Square (df), p	9.739 (4), 0.045 *				

## Discussion

###  Key results

 The findings of this study are notable in that they explore immigration status as it relates to HISB. The findings from this study indicate that immigration status is an important influencer of seeking information from a medical professional. The literature offers a number of explanations for this outcome. Massey et al^20^highlight how both foreign-born nativity and language are significant determinants of health information seeking among non-White Hispanics. Disparities in access to healthcare for immigrant populations could also explain why said population is less likely to seek health information from medical professionals,^21^given the nativity-and citizenship-based exclusions from public programs for immigrants. Similarly, Filipino domestic migrant workers in Hong Kong cited language, immigration challenges, and quality of information as barriers to information seeking and scanning behaviors.^22^ Social support was found to be an important factor related to health information seeking among Korean immigrants.^23^ Also, Korean natives and Korean American immigrants had varying degrees of trust in information sources. The social networks created a sense of belonging, attenuated language barriers, and facilitated information flow. This social support is also manifested on social media, which even facilitates the immigration process itself for certain immigrants.^24^ Variation of health information seeking sources by socioeconomic status and race are also noteworthy factors to take into account when researching HISB.

 Similar to previous research,^5^ this study found that digital HISB is prevalent among college students. This trend increased throughout the pandemic as college students used the internet and social media to acquire COVID-related health information.^25,26^ The use of online channels to acquire health information shows no signs of abating, yet college students’ ability to discern the credibility of that information remains underdeveloped.^10,25,27^While college students lack confidence in the e-health information they acquire, they readily share information with others in their network yet there is variation in seeking consultation from health providers.^10^ Thus, the primary distinctions among the research findings are in the health behaviors college students engage in after information seeking. The results of this study corroborate previous research that the likelihood for college students to confirm health information found digitally with medical health professionals varies by sociodemographic factors.^7,10^ This study is novel not only because of its racially and ethnically diverse sample, but also in identifying immigration status among urban college students as a potential determinant for HISB.

 Also, while social networks prove effective tools to disseminate health information, ensuring the accuracy of information that is exchanged is of utmost importance. This means making concerted efforts to serve people in their native languages, especially given the diversity of the United States and the likelihood of immigrants turning to sources other than medical professionals when engaged in HISB. Also, targeted educational programs designed to increase health information seeking skills, including discernment of information quality may improve health outcomes for immigrant populations. To reduce health disparities, conditions that impede marginalized individuals from accessing healthcare and engaging with healthcare professionals should be addressed. These implications are especially important given that immigrant populations are disproportionately affected by chronic disease.^28^ Given that regard of information sources may change upon immigration to a new country,^29^ future research should explore this notion in order to serve our diverse populations in New York and nationally. Additional research is needed to explore the role of perceived credibility of social media influencers in educating the public about health issues.

 This study is limited by the cross-sectional design which represents data collection at a single point in time that cannot be generalized to other populations. The responses are based on self-report. While the measurement tools were excerpted from larger validated instruments, its psychometric properties were not validated with the new population. Also, the historical factor of the pandemic may have both biased responses and reduced response rates. Finally, the sample size is relatively small to assess HISB in this cross-sectional survey.

 Despite the limitations, this study fills a gap in the literature due to the limited amount of research done on immigration status and the diverse populations represented in this sample population. Further, this is a timely contribution to the literature due to the impending COVID-19 “infodemic.” This research can serve as a foundation to explore the generalizability of these findings to other similar schools.

## Acknowledgements

 The authors thank the Collaborative Research Group on Health Policy & Promotion & UrbanHealth Lab and York College (CUNY) for their support.

## Author Contributions

**Conceptualization:** Vincent Jones II.

**Data Curation:** Sungwoo Kim.

**Formal Analysis:** Sungwoo Kim.

**Investigation:** Vincent Jones II.

**Project administration:** Shyanne Huggins.

**Supervision:** Corey Basch.

**Writing – original draft, review & editing: **Vincent Jones II, Sungwoo Kim, Apeksha Mewani, Erin T. Jacques, Mary-Andrée Ardouin-Guerrier, and Corey Basch.

## Funding

 This study received no funding.

## Ethical Approval

 This study was approved by the Institutional Review Board at York College Protocol number- 2020-0573.

## Competing Interests

 The authors declare that they have no conflict of interest.

## Supplementary Files


Supplementary file 1. Correlations.
