# Supplementary material for: Immigration status as a determinant of health information-seeking behavior among undergraduates of color at an urban commuter college
Source: Health Promot Perspect. 2022 Dec 10;12(3):295–300. doi: 10.34172/hpp.2022.38 (PMC9808907; doi:10.34172/hpp.2022.38)
Supplement: Supplementary file 1 — Correlations. [file hpp-12-295-s001.pdf]

Jones et al, **Health Promotion Perspectives**, 2022, 12(3), 295-300
doi: 10.34172/hpp.2022.38
https://hpp.tbzmed.ac.ir

Immigration status as a determinant of health information-seeking behavior among undergraduates of color at an urban commuter college

Vincent Jones II[1*], Sungwoo Kim[2], Apeksha H. Mewani[3], Erin T. Jacques[3], Mary-Andrée Ardouin-Guerrier[1], Shyanne Huggins[4], Corey H. Basch[5]

[1]Department of Health and Human Performance, York College, The City University of New York, Jamaica, NY 11451, USA
[2]Department of Human Development, Teachers College, Columbia University, New York, NY 10027, USA
[3]Department of Health and Behavior Studies, Teachers College, Columbia University, New York, NY 10027, USA
[4]Health Promotion Center, York College, The City University of New York, Jamaica, NY 11451, USA
[5]Department of Public Health, William Paterson University, Wayne, NJ 07470, USA

Heath Promotion Center and Health Information-Seeking Survey

Start of Block: Screening Questions

Q1 Are you a student at York College (CUNY)?

○ Yes (28)

○ No (29)

Q2 Did you graduate before Fall 2019?

○ Yes (23)

○ No (24)

---

*

Q3 What is your age in years?

____

Q4 Can you respond to a survey in English?

○ Yes (1)

○ No (2)

End of Block: Screening Questions

Start of Block: Demographic Questions

Q5 Are you an undergraduate or graduate student?

○ Undergraduate (1)

○ Graduate Student (2)

Q6 Are you of Hispanic, Latino, or of Spanish origin?

○ Yes (1)

○ No (2)

Q7 How would you describe yourself?

- ☐ American Indian or Alaska Native (1)
- ☐ Asian (2)
- ☐ Black or African American (3)
- ☐ Native Hawaiian or Other Pacific Islander (4)
- ☐ White (5)
- ☐ Hispanic or Latino (6)
- ☐ Other, please specify. (7) ____

Q8 Are you the first in your family to attend college in the United States?

- ○ Yes (1)
- ○ No (2)

Q9 For how many generations has your immediate family been in the United States?

- ○ 0 (1)
- ○ 1 (2)
- ○ 2 (3)
- ○ More than 2 (4)

Q10 Are you an international student?

- Yes (5)
- No (6)

Display This Question:

If Are you an undergraduate or graduate student? = Undergraduate

Q11 What Year are You?

- Freshman (1)
- Sophomore (2)
- Junior (3)
- Senior (4)

Q12 Which of the following best describes you?

- I identify as a man (1)
- I identify as a woman (2)
- I identify as gender non-binary (3)
- I identify as a transgender male\transgender man (4)
- I identify as a transgender female\transgender woman (5)
- I identify as gender queer\gender non-conforming (6)
- I have another gender identity (7)
- I prefer not to disclose (8)

Q13 Please estimate your household income.

- ◯ Less than $20,000 (2)
- ◯ $20,000 to $34,999 (3)
- ◯ $35,000 to $49,999 (5)
- ◯ $50,000 to $74,999 (6)
- ◯ $75,000 to $99,999 (7)
- ◯ Over $100,000 (8)

Q14 Are you a transfer student?

- ◯ Yes (1)
- ◯ No (2)

---

*

Q15 Including this term, how many credits have you earned?

____

---

*

Q16 How many credits are you taking this semester?

____

---

*

Q17 How many credits of fully online courses did you take last fall?

____

Q18 How many credits of hybrid courses did you take last fall?

____

Display This Question:

If Are you an undergraduate or graduate student? = Graduate Student

Q19 How much of the total coursework for your masters program have you completed?

- ◯ 25% or less (1)
- ◯ Between 26 and 50 percent (2)
- ◯ Between 51 and 75 percent (3)
- ◯ Between 76 and 100 percent (4)

Q20 What is your academic department at York College (CUNY).

- ◯ School of Arts and Sciences (1)
- ◯ Behavioral Sciences (2)
- ◯ Biology (3)
- ◯ Chemistry (4)
- ◯ Earth and Physical Sciences (5)
- ◯ English (6)
- ◯ History and Philosophy (7)
- ◯ Mathematics and Computer Science (8)
- ◯ Performing and Fine Arts (9)
- ◯ World Languages, Literatures, and Humanities (10)
- ◯ School of Business and Information Systems (11)
- ◯ Accounting and Finance (12)
- ◯ Business and Economics (13)
- ◯ CUNY Aviation Institute (14)
- ◯ School of Health Sciences and Professional Programs (15)
- ◯ Health Professions (16)
- ◯ Health & Human Performance (17)
- ◯ Nursing (18)
- ◯ Occupational Therapy (19)
- ◯ Social Work (20)
- ◯ Teacher Education (21)

Q21 Do you participate in any of these programs? (Check all that apply)

- ☐ Athletics (1)
- ☐ Clubs and Organizations (2)
- ☐ Honors Program (3)
- ☐ Scholarships (4)
- ☐ SEEK Program (5)
- ☐ Veteran Services (6)
- ☐ Army ROTC (7)

Q22 How many times per week did you commute to campus in fall 2019.

- ○ Daily (1)
- ○ 4-6 times a week (2)
- ○ 2-3 times a week (3)
- ○ Once a week (4)
- ○ Never (5)

Q23 Over fall semester, how long did it typically take you to travel to York College (CUNY) from your most common starting location?

○ 0-20 Minutes (1)

○ 21-40 Minutes (2)

○ 41-60 Minutes (3)

○ 61-90 Minutes (4)

○ More than 90 Minutes (5)

End of Block: Demographic Questions

Start of Block: Part 2: The Health Promotion Center

Q24 Are you aware of the Health Promotion Center at York College (CUNY)

○ Yes (1)

○ No (2)

Skip To: Q30 If Are you aware of the Health Promotion Center at York College (CUNY) = No

---

Q25 How many times in the past year did you visit the Health Promotion Center?

○ 1-2 (1)

○ 3-5 (2)

○ 6-8 (3)

○ More than 8 times (4)

○ Never (5)

Skip To: Q30 If How many times in the past year did you visit the Health Promotion Center? = Never

---

Q26 Did you go to the Health Promotion Center because a class obligated you to go?

- Yes (1)
- No (2)

Q27 Did you go to the Heath Promotion Center for athletic advisement?

- Yes (1)
- No (4)

Q28 Please rate your satisfaction with the following:

	Extremely satisfied (22)	Moderately satisfied (23)	Neither satisfied or dissatisfied (24)	Slightly dissatisfied (25)	Dissatisfied (26)	N/A (29)
Computers (1)	○	○	○	○	○	○
The space itself (2)	○	○	○	○	○	○
Website (3)	○	○	○	○	○	○
Printers (4)	○	○	○	○	○	○
Sexual heath items (condoms, lubricants) (5)	○	○	○	○	○	○
Informational flyers (6)	○	○	○	○	○	○
Staff's responses to your questions (7)	○	○	○	○	○	○
Screenings (BMI test) (8)	○	○	○	○	○	○

Q29 During the Spring 2019 and Fall 2019 semesters how often have you utilized each of these offerings:

	Always (1)	Most of the time (2)	About half the time (3)	Sometimes (4)	Never (5)
Computers (1)	○	○	○	○	○
Printers (2)	○	○	○	○	○
Sexual Health Items (Condoms, Lubricants) (3)	○	○	○	○	○
Informational Flyers (4)	○	○	○	○	○
Asked Health Related Questions to Staff (5)	○	○	○	○	○

Q30 I would prefer to utilize live webinars (online workshops) over in-person workshops about health topics.

○ Strongly Agree (1)

○ Agree (2)

○ Neutral (3)

○ Disagree (4)

○ Strongly Disagree (5)

Q31 Would you attend a webinar or in-person session on the following health issues?

	Extremely Likely (35)	Likely (36)	Neither likely nor unlikely (37)	Unlikely (38)	Extremely Unlikely (39)
Alcohol & other drugs (1)	o	o	o	o	o
Weight Management (2)	o	o	o	o	o
Healthy Relationships (3)	o	o	o	o	o
Domestic Violence (4)	o	o	o	o	o
Sexual Consent (6)	o	o	o	o	o
COVID-19 (7)	o	o	o	o	o
Contraception (8)	o	o	o	o	o
Men's sexual health (9)	o	o	o	o	o
Women's sexual health (10)	o	o	o	o	o
Sexually transmitted disorders (11)	o	o	o	o	o
Eating disorders (12)	o	o	o	o	o
Stress management (13)	o	o	o	o	o

End of Block: Part 2: The Health Promotion Center

Start of Block: Health Information Seeking (HIS)

Q32 Have you ever looked for information about health or medical topics from any source in the past 12 months?

- ○ Yes (1)
- ○ No (2)

Q33 In general, how often do you use each of the following sources to get health information

	Always (18)	Most of the time (19)	About half the time (20)	Sometimes (21)	Never (22)
Family and/or friends (1)	○	○	○	○	○
Health or medical professional (2)	○	○	○	○	○
Internet (3)	○	○	○	○	○
Newspaper or books (4)	○	○	○	○	○
Social media (5)	○	○	○	○	○
Television (13)	○	○	○	○	○
Community Center (14)	○	○	○	○	○
Spiritual/Religious Institution (15)	○	○	○	○	○

Q34 How many hours a day do you spend browsing the internet?

____

Q35 How many hours a day do you spend using social media?

____

Q36 In the past 12 months, have you used any source on the Internet for information about someone else's health?

- ○ Yes (1)
- ○ No (2)

Q37 When you have a health or medical question, where on the Internet do you typically go to first? (Check only one)

- ○ Everyday Health (1)
- ○ Healthline (2)
- ○ Mayo Clinic (3)
- ○ MedicineNet (4)
- ○ National Institutes of Health(NIH) (5)
- ○ WebMD (6)
- ○ Yahoo!Health (7)
- ○ Other please specify (8) ____
- ○ N\A (9)

Q38 When searching health or medical issues, how accurate do you think the information on the Internet is?

- Extremely accurate (13)
- Very accurate (14)
- Moderately accurate (15)
- Slightly accurate (16)
- Not accurate at all (17)

Q39 When searching health or medical issues on the Internet, how likely are you to gather information using several web sources to evaluate and approve its accuracy?

- Extremely likely (25)
- Somewhat likely (26)
- Neither likely nor unlikely (27)
- Somewhat unlikely (28)
- Extremely unlikely (29)

Q40 When searching health or medical issues on the Internet, how likely are you to share the information you find with family or friends without checking its accuracy?

- Extremely likely (18)
- Somewhat likely (19)
- Neither likely nor unlikely (20)
- Somewhat unlikely (21)
- Extremely unlikely (22)

Q41 When searching health or medical issues on the Internet, how likely are you to confirm the information you find with a health or medical professional?

- ○ Extremely likely (20)
- ○ Somewhat likely (21)
- ○ Neither likely nor unlikely (22)
- ○ Somewhat unlikely (23)
- ○ Extremely unlikely (24)

Q42 The number of followers of the information provider on social media influences the accuracy of the information presented

- ○ Strongly agree (16)
- ○ Agree (17)
- ○ Somewhat agree (18)
- ○ Neither agree nor disagree (19)
- ○ Somewhat disagree (20)
- ○ Disagree (21)
- ○ Strongly disagree (22)

Q43 The Internet provides helpful resources for health information

- Strongly agree (4)
- Agree (5)
- Somewhat agree (6)
- Neither agree nor disagree (7)
- Somewhat disagree (8)
- Disagree (9)
- Strongly disagree (10)

Q44 Social media provides helpful resources for health information

- Strongly agree (4)
- Agree (5)
- Somewhat agree (6)
- Neither agree nor disagree (7)
- Somewhat disagree (8)
- Disagree (9)
- Strongly disagree (10)

Q45 When encountering health or medical topics on social media, how accurate do you think the information is?

- Extremely accurate (18)
- Very accurate (19)
- Moderately accurate (20)
- Slightly accurate (21)
- Not accurate at all (22)

Q46 In the past 12 months have you self-diagnosed a medical problem?

- Yes (1)
- No (2)

Skip To: End of Survey If In the past 12 months have you self-diagnosed a medical problem? = No

---

Page Break

Q47 If you have self-diagnosed a medical problem, have you seen a medical professional to assess and confirm your condition?

- Yes (1)
- No (2)
- N/A (3)

End of Block: Health Information Seeking (HIS)
